# Ethyl 5-(4-chloro­phen­yl)-2-[(*Z*)-(methoxy­carbon­yl)methyl­ene]-7-methyl-3-oxo-3,5-dihydro-2*H*-thia­zolo[3,2-*a*]pyrimi­dine-6-carboxyl­ate

**DOI:** 10.1107/S1600536809002451

**Published:** 2009-01-23

**Authors:** Zhao-Hui Hou, Ning-Bo Zhou, Bin-Hong He, Xiao-Fang Li

**Affiliations:** a Department of Chemistry and Chemical Engineering, Hunan Institute of Science and Technology, Yueyang 414000, People’s Republic of China; b School of Chemistry and Chemical Engineering, Hunan University of Science and Technology, Xiangtan 411201, People’s Republic of China

## Abstract

The title compound, C_19_H_17_ClN_2_O_5_S, was synthesized by the reaction of ethyl 6-(4-chloro­phen­yl)-2-mercapto-4-methyl-1,6-dihydro­pyrimidine-5-carboxyl­ate and dimethyl acetyl­ene­dicarboxyl­ate in methanol. In the mol­ecule, the nearly planar thia­zole ring, with a mean deviation from the plane of 0.0108 (3) Å, is fused with a dihydro­pyrimidine ring in a flattened half-chair conformation.

## Related literature

For the biological activity of fused pyrimidine derivatives, see: Ashok *et al.* (2007 ▶); Monks *et al.* (1991 ▶). For structures containing a fused pyrimidine ring, see: Liu *et al.* (2004 ▶); Sridhar *et al.* (2006 ▶); Hou (2009 ▶).
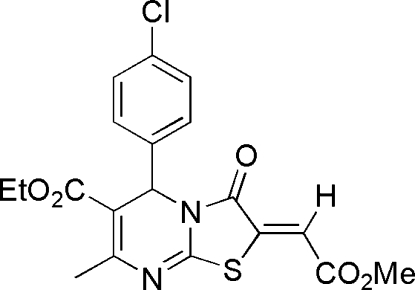


## Experimental

### 

#### Crystal data

C_19_H_17_ClN_2_O_5_S
*M* *_r_* = 420.86
Triclinic, 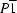

*a* = 9.6687 (19) Å
*b* = 11.052 (2) Å
*c* = 11.064 (2) Å
α = 108.04 (3)°
β = 104.70 (3)°
γ = 111.82 (3)°
*V* = 948.2 (3) Å^3^
*Z* = 2
Mo *K*α radiation
μ = 0.35 mm^−1^
*T* = 113 (2) K
0.20 × 0.14 × 0.10 mm

#### Data collection

Rigaku Saturn diffractometer
Absorption correction: multi-scan (*CrystalClear*; Rigaku, 2005 ▶) *T*_min_ = 0.934, *T*_max_ = 0.966
6937 measured reflections
3324 independent reflections
2622 reflections with *I* > 2σ(*I*)
*R*_int_ = 0.025

#### Refinement

*R*[*F* ^2^ > 2σ(*F* ^2^)] = 0.031
*wR*(*F* ^2^) = 0.090
*S* = 1.08
3324 reflections
256 parameters
H-atom parameters constrained
Δρ_max_ = 0.21 e Å^−3^
Δρ_min_ = −0.31 e Å^−3^

### 

Data collection: *CrystalClear* (Rigaku, 2005 ▶); cell refinement: *CrystalClear*; data reduction: *CrystalClear*; program(s) used to solve structure: *SHELXS97* (Sheldrick, 2008 ▶); program(s) used to refine structure: *SHELXL97* (Sheldrick, 2008 ▶); molecular graphics: *SHELXTL* (Sheldrick, 2008 ▶); software used to prepare material for publication: *SHELXTL*.

## Supplementary Material

Crystal structure: contains datablocks I, global. DOI: 10.1107/S1600536809002451/fl2231sup1.cif

Structure factors: contains datablocks I. DOI: 10.1107/S1600536809002451/fl2231Isup2.hkl

Additional supplementary materials:  crystallographic information; 3D view; checkCIF report

## Figures and Tables

**Fig. 1.**
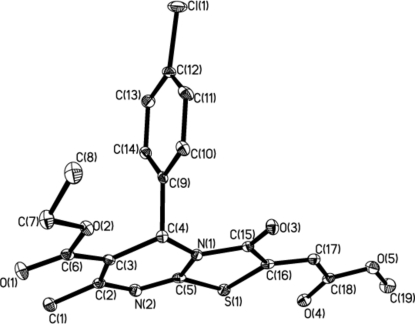
The molecular structure of (I), drawn with 30% probability ellipsoids. H atoms have been omitted for clarity.

**Fig. 2.**
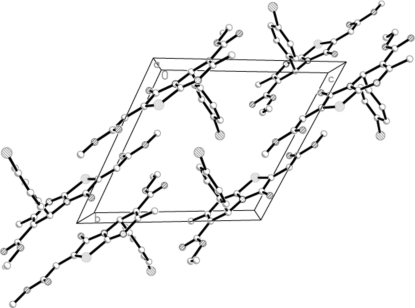
The crystal structure of (I), viewed along a axis.

## supplementary crystallographic information

### Comment

Fused pyrimidine derivatives represent important target molecules due to their highly pronounced biological properties. (Ashok *et al.*, 2007; Monks *et al.*, 1991). In this paper, the structure of the title compound (I) is reported  (Fig. 1).

The thiazole ring (C5—N1—C15—C16—S1) has the usual essentially planar geometry observed in other fused thiazolopyrimidine compounds (Liu *et al.*, 2004; Sridhar *et al.*, 2006; Hou, 2009). The thiazole ring makes a dihedral angles of 90.3 (2) ° with the benzene ring (C9–C14). The pyrimidine ring adopts a flattened half-chair conformation. The C16—C17 double bond exist in the *Z* configuration.  Molecular packing (Fig. 2) is stabilized mainly by weak van der Waals forces.

### Experimental

A mixture of ethyl 2-mercapto-4-methyl-6-(4-chlorophenyl)-1,6- dihydro-pyrimidine-5-carboxylate (0.01 mol), dimethyl acetylenedicarboxylate (0.01 mol) in methanol (25 ml) was refluxed for 3 h. The reaction mixture was cooled and filtered. The resulting solid was collected and crystallized from methanol to obtain the final product (90% yield, mp 434–435 K). ^1^H NMR (CDCl_3_, p.p.m.): 1.20 (t, J=7.0 Hz, 3H), 2.53 (s, 3H), 3.86 (s, 3H), 4.10–4.12 (m, 2H), 6.12 (s, 1H), 6.90 (s, 1H), 7.28–7.32 (m, 4H); The compound was recrystallized by slow evaporation of a methanol solution, yielding yellow, blocklike single crystals suitable for *X*–ray diffraction.

### Crystal data

**Table:**

C_19_H_17_ClN_2_O_5_S	*Z* = 2
*M**_r_* = 420.86	*F*(000) = 436
Triclinic, *P*1	*D*_x_ = 1.474 Mg m^−^^3^
*a* = 9.6687 (19) Å	Mo *K*α radiation, λ = 0.71073 Å
*b* = 11.052 (2) Å	Cell parameters from 3131 reflections
*c* = 11.064 (2) Å	θ = 2.1–27.9°
α = 108.04 (3)°	µ = 0.35 mm^−^^1^
β = 104.70 (3)°	*T* = 113 K
γ = 111.82 (3)°	Block, yellow
*V* = 948.2 (3) Å^3^	0.20 × 0.14 × 0.10 mm

### Data collection

**Table:**

Rigaku Saturn diffractometer	3324 independent reflections
Radiation source: rotating anode	2622 reflections with *I* > 2σ(*I*)
confocal	*R*_int_ = 0.025
ω scans	θ_max_ = 25.0°, θ_min_ = 2.1°
Absorption correction: multi-scan (*CrystalClear*; Rigaku, 2005)	*h* = −11→11
*T*_min_ = 0.934, *T*_max_ = 0.966	*k* = −13→13
6937 measured reflections	*l* = −11→13

### Refinement

**Table:**

Refinement on *F*^2^	Primary atom site location: structure-invariant direct methods
Least-squares matrix: full	Secondary atom site location: difference Fourier map
*R*[*F*^2^ > 2σ(*F*^2^)] = 0.031	Hydrogen site location: inferred from neighbouring sites
*wR*(*F*^2^) = 0.090	H-atom parameters constrained
*S* = 1.08	*w* = 1/[σ^2^(*F*_o_^2^) + (0.0541*P*)^2^] where *P* = (*F*_o_^2^ + 2*F*_c_^2^)/3
3324 reflections	(Δ/σ)_max_ = 0.005
256 parameters	Δρ_max_ = 0.21 e Å^−^^3^
0 restraints	Δρ_min_ = −0.31 e Å^−^^3^

### Special details

**Table:**

Geometry. All e.s.d.'s (except the e.s.d. in the dihedral angle between two l.s. planes) are estimated using the full covariance matrix. The cell e.s.d.'s are taken into account individually in the estimation of e.s.d.'s in distances, angles and torsion angles; correlations between e.s.d.'s in cell parameters are only used when they are defined by crystal symmetry. An approximate (isotropic) treatment of cell e.s.d.'s is used for estimating e.s.d.'s involving l.s. planes.
Refinement. Refinement of *F*^2^ against ALL reflections. The weighted *R*-factor *wR* and goodness of fit *S* are based on *F*^2^, conventional *R*-factors *R* are based on *F*, with *F* set to zero for negative *F*^2^. The threshold expression of *F*^2^ > σ(*F*^2^) is used only for calculating *R*-factors(gt) *etc*. and is not relevant to the choice of reflections for refinement. *R*-factors based on *F*^2^ are statistically about twice as large as those based on *F*, and *R*- factors based on ALL data will be even larger.

### Fractional atomic coordinates and isotropic or equivalent isotropic displacement parameters (Å^2^)

**Table:**

	*x*	*y*	*z*	*U*_iso_*/*U*_eq_	
S1	0.27994 (5)	0.74177 (5)	0.87884 (4)	0.01936 (13)	
Cl1	0.83796 (7)	0.55727 (6)	0.46536 (6)	0.04201 (17)	
O1	0.64107 (15)	1.21151 (13)	0.64452 (12)	0.0252 (3)	
O2	0.83679 (14)	1.16857 (12)	0.74775 (11)	0.0186 (3)	
O3	0.73679 (14)	0.84993 (13)	0.99591 (12)	0.0221 (3)	
O4	0.19590 (15)	0.58671 (13)	1.02307 (12)	0.0243 (3)	
O5	0.35078 (15)	0.50934 (13)	1.13158 (13)	0.0262 (3)	
N1	0.54957 (16)	0.88470 (15)	0.85693 (13)	0.0157 (3)	
N2	0.31742 (17)	0.89611 (16)	0.73589 (14)	0.0200 (3)	
C1	0.3241 (2)	1.0508 (2)	0.62209 (18)	0.0232 (4)	
H1A	0.3709	1.1541	0.6765	0.035*	
H1B	0.2115	1.0024	0.6075	0.035*	
H1C	0.3300	1.0314	0.5333	0.035*	
C2	0.4179 (2)	0.99530 (18)	0.69891 (16)	0.0178 (4)	
C3	0.5774 (2)	1.03422 (18)	0.73312 (16)	0.0158 (4)	
C4	0.6610 (2)	0.96835 (18)	0.80727 (17)	0.0158 (4)	
H4	0.7612	1.0482	0.8889	0.019*	
C5	0.3874 (2)	0.85167 (18)	0.81262 (16)	0.0170 (4)	
C6	0.6833 (2)	1.14651 (18)	0.70222 (16)	0.0173 (4)	
C7	0.9543 (2)	1.27881 (19)	0.72632 (19)	0.0233 (4)	
H7A	0.9846	1.3759	0.7921	0.028*	
H7B	0.9079	1.2670	0.6320	0.028*	
C8	1.1019 (2)	1.2568 (2)	0.7497 (2)	0.0328 (5)	
H8A	1.1400	1.2595	0.8401	0.049*	
H8B	1.1874	1.3330	0.7455	0.049*	
H8C	1.0723	1.1640	0.6786	0.049*	
C9	0.7070 (2)	0.86924 (18)	0.71575 (17)	0.0155 (4)	
C10	0.8681 (2)	0.89652 (19)	0.76003 (18)	0.0210 (4)	
H10	0.9482	0.9778	0.8434	0.025*	
C11	0.9108 (2)	0.8038 (2)	0.6811 (2)	0.0253 (4)	
H11	1.0189	0.8226	0.7102	0.030*	
C12	0.7895 (2)	0.6831 (2)	0.5585 (2)	0.0254 (4)	
C13	0.6299 (2)	0.6568 (2)	0.50944 (19)	0.0236 (4)	
H13	0.5509	0.5776	0.4242	0.028*	
C14	0.5891 (2)	0.75084 (18)	0.58961 (17)	0.0186 (4)	
H14	0.4818	0.7340	0.5582	0.022*	
C15	0.5986 (2)	0.82795 (18)	0.94441 (16)	0.0167 (4)	
C16	0.4553 (2)	0.73775 (18)	0.96435 (17)	0.0178 (4)	
C17	0.4656 (2)	0.66465 (18)	1.04032 (17)	0.0193 (4)	
H17	0.5632	0.6650	1.0787	0.023*	
C18	0.3229 (2)	0.58440 (18)	1.06279 (17)	0.0200 (4)	
C19	0.2160 (3)	0.4307 (2)	1.1621 (2)	0.0341 (5)	
H19A	0.2063	0.4991	1.2332	0.051*	
H19B	0.2374	0.3656	1.1945	0.051*	
H19C	0.1156	0.3756	1.0787	0.051*	

### Atomic displacement parameters (Å^2^)

**Table:**

	*U*^11^	*U*^22^	*U*^33^	*U*^12^	*U*^13^	*U*^23^
S1	0.0146 (2)	0.0231 (3)	0.0214 (2)	0.0090 (2)	0.00922 (19)	0.0103 (2)
Cl1	0.0664 (4)	0.0408 (3)	0.0612 (4)	0.0422 (3)	0.0514 (3)	0.0323 (3)
O1	0.0212 (7)	0.0253 (7)	0.0322 (7)	0.0122 (6)	0.0078 (6)	0.0180 (6)
O2	0.0143 (6)	0.0174 (6)	0.0248 (6)	0.0073 (5)	0.0076 (5)	0.0112 (5)
O3	0.0144 (7)	0.0281 (7)	0.0257 (7)	0.0108 (6)	0.0070 (5)	0.0152 (6)
O4	0.0203 (7)	0.0257 (7)	0.0259 (7)	0.0094 (6)	0.0118 (6)	0.0108 (6)
O5	0.0321 (8)	0.0242 (7)	0.0363 (7)	0.0160 (7)	0.0232 (7)	0.0200 (6)
N1	0.0134 (8)	0.0181 (8)	0.0170 (7)	0.0084 (7)	0.0066 (6)	0.0083 (6)
N2	0.0156 (8)	0.0245 (9)	0.0232 (8)	0.0119 (7)	0.0087 (6)	0.0115 (7)
C1	0.0180 (10)	0.0290 (11)	0.0252 (9)	0.0152 (9)	0.0066 (8)	0.0127 (8)
C2	0.0195 (10)	0.0186 (9)	0.0140 (8)	0.0110 (8)	0.0062 (7)	0.0045 (7)
C3	0.0169 (9)	0.0153 (9)	0.0147 (8)	0.0099 (8)	0.0053 (7)	0.0047 (7)
C4	0.0120 (9)	0.0165 (9)	0.0173 (8)	0.0058 (7)	0.0054 (7)	0.0079 (7)
C5	0.0129 (9)	0.0164 (9)	0.0173 (8)	0.0066 (8)	0.0064 (7)	0.0032 (7)
C6	0.0175 (9)	0.0162 (9)	0.0150 (8)	0.0092 (8)	0.0049 (7)	0.0037 (7)
C7	0.0163 (10)	0.0206 (10)	0.0287 (10)	0.0040 (8)	0.0071 (8)	0.0143 (8)
C8	0.0212 (11)	0.0423 (13)	0.0442 (12)	0.0149 (10)	0.0168 (9)	0.0284 (11)
C9	0.0165 (9)	0.0172 (9)	0.0204 (9)	0.0103 (8)	0.0101 (7)	0.0130 (7)
C10	0.0167 (10)	0.0217 (10)	0.0292 (10)	0.0099 (8)	0.0106 (8)	0.0152 (8)
C11	0.0205 (10)	0.0301 (11)	0.0445 (12)	0.0179 (9)	0.0220 (9)	0.0253 (10)
C12	0.0403 (12)	0.0271 (11)	0.0373 (11)	0.0257 (10)	0.0311 (10)	0.0240 (9)
C13	0.0311 (11)	0.0199 (10)	0.0236 (9)	0.0137 (9)	0.0141 (8)	0.0104 (8)
C14	0.0175 (10)	0.0200 (9)	0.0211 (9)	0.0102 (8)	0.0086 (8)	0.0110 (8)
C15	0.0192 (10)	0.0156 (9)	0.0143 (8)	0.0095 (8)	0.0067 (7)	0.0048 (7)
C16	0.0160 (9)	0.0166 (9)	0.0168 (8)	0.0077 (8)	0.0075 (7)	0.0027 (7)
C17	0.0196 (10)	0.0185 (9)	0.0190 (9)	0.0100 (8)	0.0081 (8)	0.0064 (7)
C18	0.0235 (10)	0.0166 (9)	0.0165 (9)	0.0090 (8)	0.0091 (8)	0.0040 (7)
C19	0.0415 (13)	0.0302 (12)	0.0459 (12)	0.0164 (11)	0.0317 (11)	0.0252 (10)

### Geometric parameters (Å, °)

**Table:**

S1—C16	1.7436 (17)	C7—C8	1.507 (2)
S1—C5	1.7582 (17)	C7—H7A	0.9700
Cl1—C12	1.7484 (18)	C7—H7B	0.9700
O1—C6	1.2084 (18)	C8—H8A	0.9600
O2—C6	1.338 (2)	C8—H8B	0.9600
O2—C7	1.4556 (19)	C8—H8C	0.9600
O3—C15	1.2063 (19)	C9—C14	1.383 (3)
O4—C18	1.208 (2)	C9—C10	1.389 (2)
O5—C18	1.3390 (19)	C10—C11	1.388 (2)
O5—C19	1.460 (2)	C10—H10	0.9300
N1—C5	1.380 (2)	C11—C12	1.380 (3)
N1—C15	1.385 (2)	C11—H11	0.9300
N1—C4	1.4804 (19)	C12—C13	1.380 (3)
N2—C5	1.278 (2)	C13—C14	1.390 (2)
N2—C2	1.415 (2)	C13—H13	0.9300
C1—C2	1.506 (2)	C14—H14	0.9300
C1—H1A	0.9600	C15—C16	1.492 (2)
C1—H1B	0.9600	C16—C17	1.344 (2)
C1—H1C	0.9600	C17—C18	1.467 (2)
C2—C3	1.348 (2)	C17—H17	0.9300
C3—C6	1.484 (2)	C19—H19A	0.9600
C3—C4	1.525 (2)	C19—H19B	0.9600
C4—C9	1.525 (2)	C19—H19C	0.9600
C4—H4	0.9800		
			
C16—S1—C5	90.62 (8)	C7—C8—H8C	109.5
C6—O2—C7	116.24 (12)	H8A—C8—H8C	109.5
C18—O5—C19	115.21 (14)	H8B—C8—H8C	109.5
C5—N1—C15	115.79 (14)	C14—C9—C10	119.53 (15)
C5—N1—C4	121.86 (13)	C14—C9—C4	120.55 (14)
C15—N1—C4	122.26 (13)	C10—C9—C4	119.91 (16)
C5—N2—C2	116.82 (14)	C11—C10—C9	120.70 (18)
C2—C1—H1A	109.5	C11—C10—H10	119.7
C2—C1—H1B	109.5	C9—C10—H10	119.7
H1A—C1—H1B	109.5	C12—C11—C10	118.56 (16)
C2—C1—H1C	109.5	C12—C11—H11	120.7
H1A—C1—H1C	109.5	C10—C11—H11	120.7
H1B—C1—H1C	109.5	C11—C12—C13	121.80 (16)
C3—C2—N2	122.50 (14)	C11—C12—Cl1	119.10 (14)
C3—C2—C1	126.35 (16)	C13—C12—Cl1	119.07 (16)
N2—C2—C1	111.13 (14)	C12—C13—C14	118.87 (18)
C2—C3—C6	121.48 (14)	C12—C13—H13	120.6
C2—C3—C4	122.65 (15)	C14—C13—H13	120.6
C6—C3—C4	115.87 (14)	C9—C14—C13	120.43 (16)
N1—C4—C3	108.32 (12)	C9—C14—H14	119.8
N1—C4—C9	109.42 (13)	C13—C14—H14	119.8
C3—C4—C9	113.84 (12)	O3—C15—N1	124.33 (15)
N1—C4—H4	108.4	O3—C15—C16	126.19 (14)
C3—C4—H4	108.4	N1—C15—C16	109.48 (13)
C9—C4—H4	108.4	C17—C16—C15	122.53 (15)
N2—C5—N1	126.49 (15)	C17—C16—S1	125.85 (14)
N2—C5—S1	121.06 (13)	C15—C16—S1	111.62 (11)
N1—C5—S1	112.42 (11)	C16—C17—C18	120.08 (15)
O1—C6—O2	123.10 (15)	C16—C17—H17	120.0
O1—C6—C3	126.17 (15)	C18—C17—H17	120.0
O2—C6—C3	110.73 (13)	O4—C18—O5	124.64 (15)
O2—C7—C8	106.32 (13)	O4—C18—C17	123.25 (15)
O2—C7—H7A	110.5	O5—C18—C17	112.11 (14)
C8—C7—H7A	110.5	O5—C19—H19A	109.5
O2—C7—H7B	110.5	O5—C19—H19B	109.5
C8—C7—H7B	110.5	H19A—C19—H19B	109.5
H7A—C7—H7B	108.7	O5—C19—H19C	109.5
C7—C8—H8A	109.5	H19A—C19—H19C	109.5
C7—C8—H8B	109.5	H19B—C19—H19C	109.5
H8A—C8—H8B	109.5		
			
C5—N2—C2—C3	4.8 (2)	C3—C4—C9—C14	−56.3 (2)
C5—N2—C2—C1	−173.86 (14)	N1—C4—C9—C10	−114.05 (16)
N2—C2—C3—C6	−175.50 (14)	C3—C4—C9—C10	124.61 (16)
C1—C2—C3—C6	2.9 (3)	C14—C9—C10—C11	−1.9 (2)
N2—C2—C3—C4	3.6 (2)	C4—C9—C10—C11	177.13 (13)
C1—C2—C3—C4	−178.02 (15)	C9—C10—C11—C12	−0.7 (2)
C5—N1—C4—C3	12.4 (2)	C10—C11—C12—C13	3.4 (2)
C15—N1—C4—C3	−171.19 (13)	C10—C11—C12—Cl1	−174.78 (11)
C5—N1—C4—C9	−112.22 (16)	C11—C12—C13—C14	−3.3 (2)
C15—N1—C4—C9	64.19 (19)	Cl1—C12—C13—C14	174.86 (12)
C2—C3—C4—N1	−11.4 (2)	C10—C9—C14—C13	2.0 (2)
C6—C3—C4—N1	167.76 (13)	C4—C9—C14—C13	−177.04 (13)
C2—C3—C4—C9	110.58 (18)	C12—C13—C14—C9	0.5 (2)
C6—C3—C4—C9	−70.30 (19)	C5—N1—C15—O3	−177.63 (15)
C2—N2—C5—N1	−3.7 (2)	C4—N1—C15—O3	5.8 (2)
C2—N2—C5—S1	174.06 (11)	C5—N1—C15—C16	2.09 (19)
C15—N1—C5—N2	177.49 (15)	C4—N1—C15—C16	−174.52 (13)
C4—N1—C5—N2	−5.9 (3)	O3—C15—C16—C17	−2.8 (3)
C15—N1—C5—S1	−0.44 (18)	N1—C15—C16—C17	177.46 (15)
C4—N1—C5—S1	176.18 (11)	O3—C15—C16—S1	176.86 (14)
C16—S1—C5—N2	−179.16 (14)	N1—C15—C16—S1	−2.86 (16)
C16—S1—C5—N1	−1.10 (13)	C5—S1—C16—C17	−178.09 (16)
C7—O2—C6—O1	1.0 (2)	C5—S1—C16—C15	2.24 (12)
C7—O2—C6—C3	−178.78 (13)	C15—C16—C17—C18	176.57 (14)
C2—C3—C6—O1	−0.9 (3)	S1—C16—C17—C18	−3.1 (2)
C4—C3—C6—O1	180.00 (15)	C19—O5—C18—O4	−2.1 (2)
C2—C3—C6—O2	178.93 (14)	C19—O5—C18—C17	177.95 (14)
C4—C3—C6—O2	−0.21 (19)	C16—C17—C18—O4	−4.6 (3)
C6—O2—C7—C8	−165.55 (14)	C16—C17—C18—O5	175.35 (15)
N1—C4—C9—C14	65.01 (17)		
